# Effect of ribociclib on productivity losses due to breast cancer in young women in Brazil

**DOI:** 10.11606/s1518-8787.2022056004160

**Published:** 2022-11-18

**Authors:** Anna Maria Buehler, Pierre-Alexandre Dionne, David Chandiwana, Purnima Pathak, Ebuwa Igho-Osagie

**Affiliations:** I Novartis Biociências S.A. São Paulo SP Brasil Novartis Biociências S.A. São Paulo, SP, Brasil; II Novartis Global East Hanover NJ USA Novartis Global. East Hanover, NJ, USA; III Novartis Global Business Flagship Programs East Hanover NJ USA Novartis Global, Business Flagship Programs. East Hanover, NJ, USA

**Keywords:** Women, Premenopause, Breast Neoplasms, therapy, Absenteeism, Workforce, economics

## Abstract

**OBJETIVE:**

To evaluate the effect of ribociclib *versus* endocrine therapy on productivity losses due to advanced breast cancer.

**METHODS:**

Productivity data from the MONALEESA-7 trial, obtained from the results of the application of the Work Productivity and Activity Impairment (WPAI) questionnaire on progression-free survival state (43-month follow-up), were extrapolated to the 10,936 Brazilian prevalent cases of premenopausal women with hormone receptor positive/human epidermal growth factor receptor 2 negative (HR+/HER2-) breast cancer. Productivity loss was determined by quantifying the economic costs of workforce dropout over time in both treatment arms and by discounting the economic costs of absenteeism and presenteeism from workforce retention. A human capital approach was used.

**RESULTS:**

Net productivity gains in the ribociclib arm were estimated at USD 4,285,525.00, representing 316,609 added work hours over 43 months and a mean of 2,009 added work weeks per year.

**CONCLUSIONS:**

The phase III MONALEESA-7 trial productivity results applied to the Brazilian premenopausal prevalent cases of hormone receptor positive/human epidermal growth factor receptor 2 negative (HR+/HER2-) breast cancer showed that treatment with ribociclib + endocrine therapy improves workforce participation compared with endocrine therapy alone in premenopausal women with hormone receptor positive/human epidermal growth factor receptor 2 negative (HR+/HER2-) metastatic breast cancer, with potential economic gains for the Brazilian society.

## INTRODUCTION

Breast cancer is the most common type of malignancy in women, accounting for 24.5% of all women^1^. Its diagnosis is histological and typically described by the presence or absence of particular molecular receptors. Approximately 70% of breast cancers express estrogen receptor alpha (ER-α) or progesterone receptor (PR) and are referred to as hormone receptor positive (HR+)^2^. Between 10% and 20% overexpress or amplify human epidermal growth factor receptor 2 (HER2) and are referred to as HER2 positive (HER2+)^2^. Those that do not express ER-α, PR, or HER2+ are referred to as triple-negative^2^. Roughly 70% to 75% of all breast cancers are both HR+ and HER2 negative (HER2-). They are the most common type of cancer and often referred to as HR+/HER2-^2^.

Although breast cancer mortality has declined in high-income countries in the past 20 to 30 years^3^, it remains the worldwide leading cause of cancer death among women. Breast cancers become increasingly life-threatening as they progress to metastasis. Despite innovative therapies, about 30% of women with breast cancer will develop metastasis^4^. Five-year survival in patients with early disease ranges from 94% to 99%^2^, but patients with metastasis tend to have a median survival as low as one year^2^. Breast cancer has a worse prognosis among premenopausal women. Tumors tend to be more aggressive and invasive and are associated with higher recurrence and mortality^5^. A study showed that premenopausal women with breast cancer, aged ≤ 40 years, had a 40% higher mortality risk than older women^6^, and women aged ≤ 40 years with HR+/HER2- breast cancer had a two-fold higher mortality risk than older women with the same type of disease^6^.

### Productivity Burden

Women with breast cancer experience pain, fatigue, and mental health deterioration that decrease quality of life and reduce work-related productivity and participation in the workforce^7^. Besides productivity losses due to premature mortality, breast cancer contributes to significant direct and indirect costs to society. Reyes et al.^8^ estimated the mean annual costs of health care services for nonmetastatic and metastatic breast cancer to be USD $78,560 (SD = USD $95,874) and USD $183,337 (SD = USD $151,412), respectively, in the 2014 exchange rate^8^. Women with breast cancer are also more likely to lose their jobs^7^, increasing the economic burden of treatment costs placed on society. Metastatic breast cancer places a larger burden on society than nonmetastatic disease. Yin et al.^7^ observed that women with metastasis had more missed work hours (101 *versus* 84 hours; p < 0.001) per person-quarter, were 35% less likely to be employed than women with localized disease, and, when employed, caused a mean work productivity loss of USD $30,666 per year, which is USD $6,500 more than the cost associated with lost work time of women with local or locally advanced disease^7^.

### Health-related Quality of Life

Besides economic losses, young women with metastatic breast cancer experience a significant decrease in health-related quality of life due to reproductive health challenges^5^. With systemic treatment, many women express concerns about fertility and experience a sudden onset of menopause caused by ovarian suppression^5^. They show concerns about body image and sexuality, and those who plan to have children fear how pregnancy might affect their risk of recurrence^9^. Because younger premenopausal women are likely to be in the workforce, their clinical condition or treatment can interrupt their careers and have a lasting negative effect on household income. These concerns about sexual and reproductive health, career and work, job insecurity and the uncertainty of disease recurrence indicate lower health-related quality of life and greater financial burden among younger women compared with older postmenopausal women with the same disease^10^.

### Metastatic Breast Cancer in Brazil

Incidence of breast cancer is increasing in Brazil. A 74% increase in prevalence of metastasis was noted between 2008–2018^11^. The prevalence of metastatic breast cancer is 41 per 100,000 women, with a median overall survival (OS) after metastasis diagnosis of approximately 26.2 months^11^. Affected women have a mean five-year survival rate of 9%^11^. Besides mortality effects, the indirect costs of breast cancer in Brazil are substantial. Pearce et al.^12^ estimated annual productivity losses of US$350 million, in the 2012 exchange rate, from breast cancer mortality, which is the second highest economic effect among all invasive forms of cancer^12^. Alexandre et al.^13^ estimated annual productivity losses due to absenteeism among Brazilian women with metastatic breast cancer to be BRL 23.2 million. Losses from premature mortality were estimated to be BRL 1.37 billion and worth 15 productivity years^13^.

### Treatment of Metastatic Breast Cancer with Ribociclib

Current clinical guidelines recommend using a CDK4/6 inhibitor combined with endocrine therapy as first-line treatment for metastatic breast cancer^11^. CDK4/6 inhibitors treat cancer by targeting cyclin-dependent kinases 4 and 6 (CDK4 and CDK6) to trigger cell arrest and halt cell cycle progression. They are essential to treat metastatic breast cancer due to their ability to improve progression-free survival (PFS) and OS in women with metastatic breast cancer, and due to their low levels of toxicity^14^.

Ribociclib is an orally available CDK4/6 inhibitor that received regulatory approval from the U.S. Food and Drug Administration (FDA) for the treatment of HR+/HER2- advanced or metastatic breast cancer in postmenopausal women in August 2016. In 2018, this therapy was expanded to treat premenopausal women with metastatic breast cancer (combined with tamoxifen or an aromatase inhibitor). In Brazil, ribociclib was approved for all its indications by the *Agência Nacional de Vigilância Sanitária* (ANVISA – National Health Surveillance Agency) in July 2018. Ribociclib showed a significant effect on PFS and OS^14^. Patients receiving ribociclib combined with fulvestrant showed longer PFS than those receiving fulvestrant alone (median, 20.5 *versus* 12.8 months; hazard ratio, 0.59; 95% confidence interval, 0.48 to 0.73; p < 0.001)^15^, and 28% lower mortality risk^16^. We observed a 29% mortality reduction in the ribociclib arm (p = 0.00973)^17^ in the MONALEESA-7 trial with premenopausal and perimenopausal women. A total of 70.2% of the patients randomized to ribociclib + endocrine therapy were alive at 42 months compared with 46.0% of the patients alive in the endocrine therapy alone group^18^.

Considering its efficacy, the inaccessibility to ribociclib in Brazil will lead to higher mortality and impaired quality of life in patients with metastatic breast cancer. Reinert et al.^19^ estimated that administering ribociclib combined with endocrine therapy to one-year incident cases of HR+/HER2- metastatic breast cancer in Brazil would prevent 538 deaths over six years, compared with endocrine therapy alone. Our study complements Reinert et al.^19^ by estimating productivity gains that would benefit the Brazilian society due to access to ribociclib.

## METHODS

The study was exempted from the research ethics committee approval due to the secondary nature of the data used for analysis. We did not have access to any information allowing the identification of patients, only to the results of the questionnaires.

We extrapolated productivity data from the MONALEESA-7 trial to a 2020 prevalent HR+/HER2- metastatic breast cancer cohort in Brazil and estimated productivity gains that would benefit premenopausal women receiving ribociclib as first-line therapy for HR+/HER2- metastatic breast cancer. Productivity losses were defined as the result of permanent workforce dropout minus productivity losses due to absenteeism and presenteeism. These estimates were obtained from the results of the application of the Work Productivity and Activity Impairment (WPAI) questionnaire, as recommended by the second panel on cost-effectiveness in health and medicine^20^. Our analysis was based on the summarized individual patient data of MONALEESA-7 trial and was limited to the progression-free period, i.e., for as long as patients continued to receive ribociclib or placebo.

### MONALEESA-7 Trial

MONALEESA-7 was an international, randomized, double-blind, placebo-controlled, phase III trial that compared ribociclib with placebo, combined with tamoxifen or a nonsteroidal aromatase inhibitor (NSAI) and goserelin, as first-line therapy in premenopausal patients with HR+/HER2- advanced breast cancer^18^. The primary objective was to assess PFS in both comparator arms and the secondary was OS. The trial examined work productivity and activity impairment as secondary objectives. Table 1 shows the clinical and demographic characteristics of study participants.

**Table 1:** Demographic and clinical characteristics of participants in the MONALEESA-7 trial.

Variable		Ribociclib + ET (n = 335)	Placebo + ET (n = 337)	Total (n = 672)
Age (years), median (min-max)		43 (25–58)	45 (25–58)	44 (25–58)
Race/skin color				
	Asian	99 (29.6)	99 (29.4)	198 (29.5)
	Black	10 (3.0)	9 (2.7)	19 (2.8)
	White	187 (55.8)	201 (59.6)	388 (57.7)
	Other	39 (11.6)	28 (8.3)	67 (10.0)
ECOG performance status – n (%) at baseline				
	0	245 (73.1)	255 (75.7)	500 (74.4)
	1	87 (26.0)	78 (23.1)	165 (24.6)
	2	0 (0.0)	1 (0.3)	1 (0.3)
	Missing	3 (0.9)	3 (0.9)	6 (0.9)
Disease status at study entry				
	Locally advanced	1 (0.3)	1 (0.3)	2 (0.3)
	Distally metastatic	334 (99.7)	336 (99.7)	670 (99.7)
Hormone receptor status				
	Estrogen receptor positive	331 (99.0)	335 (99.0)	666 (99.0)
	Progesterone receptor positive	290 (87.0)	288 (85.0)	578 (86.0)
Previous surgery				
	Yes	202 (60.6)	194 (58.2)	396 (59.4)
	No	133 (39.9)	143 (43.5)	276 (41.4)
Previous radiotherapy				
	Yes	161 (48.3)	183 (54.9)	344 (51.6)
	No	174 (52.2)	154 (46.2)	328 (49.2)
Previous chemotherapy				
	For advanced disease	47 (14.0)	47 (13.9)	94 (14.0)
	Neoadjuvant or adjuvant only	138 (41.2)	138 (41.0)	276 (41.1)
	None	150 (44.8)	152 (45.1)	302 (45.0)
Disease-free interval^a^				
	Newly diagnosed disease	136 (40.6)	141 (40.4)	267 (40.2)
	Existing disease	199 (59.4)	203 (60.6)	402 (59.8)
	≤ 12 months	23 (6.9)	13 (3.9)	40 (5.9)
	> 12 months	176 (52.5)	190 (56.7)	366 (54.0)
Work productivity and activity impairment				
	Participants in the workforce at baseline (%)	35.7	40.4	38.1
	Presenteeism at baseline (%)	48.0	46.2	47.1
	Absenteeism at baseline (%)	28.4	24.9	26.7

ET: endocrine therapy; ECOG: Eastern Cooperative Oncology Group.

a Newly diagnosed disease: participants without a history of first recurrence or progression, or without first recurrence or progression within 90 days of diagnosis.

Disease-free interval for patients with existing disease was defined as the time from initial diagnosis to first recurrence or progression^17^.

### Participants

The study population included premenopausal or perimenopausal women aged 18-59 years diagnosed with HR+/HER2- advanced breast cancer who had not received prior hormone therapy and had adequate bone marrow and organ function. Women with inflammatory breast disease, central nervous system metastases, HIV, or other severe chronic conditions were excluded from the study.

### Treatment

Patients were randomly assigned in a 1:1 ratio to one of the following treatment arms: ribociclib arm – patients received ribociclib + tamoxifen or an NSAI (letrozole or anastrozole) + goserelin; placebo arm – patients received placebo + tamoxifen or an NSAI (letrozole or anastrozole) + goserelin. According to the study protocol, participants continued to receive the study medicines until disease progression (or death).

### The Work Productivity and Activity Impairment (WPAI) Questionnaire

The WPAI questionnaire is a validated patient-reported outcome instrument used to measure the following productivity components: workforce participation, absenteeism, presenteeism, and impairment in daily life activities^21^. Details of questionnaire items and scoring systems have been covered elsewhere^21^. In this study, the WPAI questionnaire was administered at treatment initiation/screening and bi-monthly to participants who remained progression free.

### Productivity Estimation

Productivity loss was estimated by quantifying the economic costs of workforce dropout over time in both treatment arms and by discounting the economic costs of absenteeism and presenteeism from workforce retention. A human capital approach was used to estimate productivity losses. This approach assumes that productivity contributions to society lost due to illness or death are irreplaceable^20^. Therefore, productivity loss is estimated as the cost of an individual's work when they are out of the workforce, regardless of replacement by substitute workers. The human capital approach is recommended for productivity estimation by the second panel on cost-effectiveness in health and medicine^20^.

Because the WPAI questionnaire was administered to participants at baseline and bi-monthly thereafter, it was possible to track workforce participation during the treatment period. Workforce dropout over time was defined as the percentage reduction of formally employed participants at baseline. Absenteeism was defined as the percentage of time formally employed participants spent away from work due to illness. Presenteeism was defined as the percentage of time formally employed participants spent away from work tasks while at work due to illness^20^. Absenteeism, presenteeism and workforce dropout figures from the MONALEESA-7 trial were subsequently extrapolated to a premenopausal population-based^11^ cohort of the prevalence of women with HR+/HER2- metastatic breast cancer in Brazil in 2020, representing 39% of breast cancer prevalent cases in Brazil (n = 10,936)^19^. All costs were estimated in Brazilian reals and converted to 2020 U.S. dollars at the prevailing exchange rate of USD $1 = BRL 5.3, obtained from the Brazilian Central Bank website calculator^22^.

### Statistical Analysis

The annual costs of workforce dropout over time in both treatment arms were estimated by multiplying a worker's payment in Brazil by the extrapolated number of patients who would leave the workforce over time. The 44-hour work per week established in Brazil and a mean daily wage rate of USD $13.54 were applied. The mean daily wage rate was estimated based on the results from the first quarter of 2020 reported by the National Household Sample Survey^23^. The monetary value of productivity losses due to absenteeism was determined by multiplying the number of missed workdays in each month by the mean daily wage rate in Brazil and by the extrapolated number of formally employed patients in each treatment arm. Presenteeism was estimated by discounting the economic value of workdays from the proportion of time of each workday spent not working at the workplace due to illness.

## RESULTS

At baseline in the MONALEESA-7 trial, 35.7% and 40.4% of women in the ribociclib and placebo arms, respectively, reported being formally employed. We applied the 38.1% mean of this sample to estimate the proportion of women with HR+/HER2- metastatic breast cancer in Brazil who participated in the workforce. By the end of the study (median of 43 months), 12.8% and 6.0% of participants in the respective treatment arms remained progression free. Figure 1 shows workforce dropout over time in the MONALEESA-7 trial.

### Extrapolation to a Brazilian Prevalence Cohort

We applied the rate of workforce dropout obtained in the MONALEESA-7 trial to a Brazilian population-based cohort of 10,936 premenopausal women with HR+/HER2- metastatic breast cancer^11,19^. Table 2 shows the results over the treatment period in both comparator arms. Productivity losses due to workforce dropout over the 43-month treatment period were estimated at USD $111,443,068.85 for women treated with ribociclib + endocrine therapy and $124,822,445.64 for those treated with placebo + endocrine therapy, which results in productivity gains of USD $13,379,376.79 in favor of treatment with add-on ribociclib. However, productivity losses due to absenteeism and presenteeism were higher in the ribociclib arm. Estimated costs for absenteeism were USD $12,423,315.29 in the ribociclib arm and USD $7,133,598.11 in the placebo arm, and USD $11,295,708.67 and USD $7,491,574.06, respectively, for presenteeism. In total, net productivity gains of USD $4,285,525.00 were achieved in the ribociclib arm, and this would result in 316,609 added work hours over 43 months – a mean of 2,009 added work weeks per year (Table 3).

**Table 2:** Breakdown of productivity losses in U.S. dollars^a^ by treatment arm extrapolated to a Brazilian population-based cohort of 10,936 women with HR+/HER2- metastatic breast cancer.

Productivity component	Ribociclib + ET (USD)	Placebo + ET (USD)	Difference (USD) (Ribo – placebo)
Workforce dropout	111,443,068.85	124,822,445.64	−13,379,376.79
Absenteeism	12,423,315.29	7,133,598.11	3,616,361.53
Presenteeism	11,295,708.67	7,491,574.06	2,600,729.98
Total	135,162,092.81	139,447,617.81	−4,285,525.00

ET: endocrine therapy.

a All costs were estimated in Brazilian reals and converted to 2020 U.S. dollars at the prevailing exchange rate of USD 1 = BRL 5.3, obtained from the Brazilian Central Bank website calculator^23^.

**Table 3:** Breakdown of net productivity gains in U.S. dollars^a^ in the ribociclib arm extrapolated to a Brazilian population-based cohort of 10,936 women with HR+/HER2- metastatic breast cancer.

Productivity variable	Estimate
Net productivity gain	USD $4,285,525.00
Difference per year	USD $1,195,960.47
Number of weeks gained over a 43-month treatment period	7,195.66 weeks
Number of weeks gained each year	2,008.09 weeks
Number of hours gained over a 43-month treatment period	316,609.04 hours
Number of hours gained each year	88,356.01 hours

a All costs were estimated in Brazilian reals and converted to 2020 U.S. dollars at the prevailing exchange rate of USD 1 = BRL 5.3, obtained from the Brazilian Central Bank website calculator^23^.

## DISCUSSION

This study assessed productivity gains from treating premenopausal women with HR+/HER2- metastatic breast cancer in Brazil with ribociclib. The results showed that women treated with ribociclib remained in the workforce at a higher rate than women treated with endocrine therapy alone, and this could lead to productivity gains of USD $13,379,376.79 over 43 months. Productivity losses associated with metastatic breast cancer in younger women must be measured, and previous studies report the negative effect of breast cancer on productivity^7,8,12,13^. Productivity losses from metastatic breast cancer place a significant economic burden on patients and on society in general. Women with advanced breast cancer cause a mean of USD $6,166 (SD, USD $9,194) in short-term disability costs per year, compared with only USD $558 (SD, USD $2,487) for healthy controls^24^. Short-term disability costs are 47% higher in women with metastatic breast cancer than in those with early breast disease^24^. In the United States, total productivity loss from missed home and work productivity was estimated at U$313 million per year, in the 2015 exchange rate, in a population-based cohort of women aged 18-64 years, while productivity loss from years of potential life lost was USD $5.7 billion^25^. A recent study estimating social costs due to work productivity loss, with ribociclib + endocrine therapy among premenopausal women with HR+/HER2- advanced breast cancer, has also been conducted from the Brazilian perspective. In that study, the same data of MONALEESA-7 trial were used but extrapolated to women at the retirement age of 62 years. Thus, the study showed that ribociclib + endocrine therapy would potentially yield BRL 71.61 million less social costs than endocrine therapy alone^26^.

Results from this study complement existing evidence on the suitability and importance of ribociclib as an add-on treatment. Treatment with ribociclib can benefit premenopausal women, as well as all women with HR+/HER2- advanced breast cancer and in first and second lines of treatment. In studies of postmenopausal women, MONALEESA-2^27^ and MONALEESA-3^15,16^, the addition of ribociclib to endocrine therapy promoted significant gains in PFS and OS, with maintenance of health-related quality of life. Moreover, in premenopausal women, the MONALEESA-7 trial showed that ribociclib + an aromatase inhibitor benefitted patients’ quality of life, decreasing the time to deterioration of quality of life and pain by > 10%^28^. These findings indicate that treatment with ribociclib may substantially improve social value and emphasize the need for new, innovative medicines to improve the quality of life of women with HR+/HER2- advanced breast cancer, as well as to prolong OS. Therefore, ribociclib reaches 100% of the objectives of a palliative treatment by prolonging patients’ life, improved quality of life, and postponing the need for chemotherapy. This clinical differentiation of ribociclib compared to other CDK4/6 inhibitors was recognized by the European Society for Medical Oncology (ESMO), in which ribociclib was the only CDK4/6 inhibitor that reached a maximum score of 5/5 on the clinical benefit magnitude scale in its latest guideline update^29^.

The estimates from this study are considered conservative. Our estimates were limited to the progression-free period aligned with the primary objective of the MONALESSA-7 trial^18,28^. Since ribociclib significantly prolongs OS compared to endocrine therapy alone, our study ignores productivity gains that would benefit from years of potential life lost and net productivity gains that would benefit in the post-progression period. By focusing only on the progression-free period, our study adopts a national payer and social perspective to answer how much the Brazilian society would benefit from treatment with ribociclib. It also addresses questions on whether clinical end points, such as PFS, are meaningful to patients’ experiences and daily functioning. Studies further exploring the association between clinical end points and quality of life in women treated with ribociclib are underway.

To our knowledge, the MONALEESA-7 trial is the first to monitor patients’ employment status and work functioning prospectively for an extended period. The WPAI questionnaire was administered monthly for 43 months, and we observed high completion rates in the trial: 99% at baseline and 98% at the end of treatment. This shows the acceptability of the WPAI questionnaire by patients and support the arguments by Basch^30^ that patients with metastatic cancer are willing to provide patient-reported outcome information during clinical trials^30^.

## CONCLUSION

The phase III MONALEESA-7 trial productivity results applied to the Brazilian premenopausal prevalent cases showed that treatment with ribociclib + endocrine therapy improves workforce participation compared with endocrine therapy alone in premenopausal women with HR+/HER2- metastatic breast cancer. Besides significantly prolonging the life of premenopausal patients with advanced breast cancer, improving their quality of life, workforce retention due to ribociclib yields economic productivity gains to society, thus supporting its use combined with endocrine therapy for premenopausal women with HR+/HER2- metastatic breast disease.
